# Analysis of drug efficacy for inflammatory skin on an organ-chip system

**DOI:** 10.3389/fbioe.2022.939629

**Published:** 2022-09-02

**Authors:** Qianghua Quan, Ding Weng, Xuan Li, Quan An, Yang Yang, Bowen Yu, Yuan Ma, Jiadao Wang

**Affiliations:** ^1^ State Key Laboratory of Tribology, Tsinghua University, Beijing, PR, China; ^2^ East Asia Skin Health Research Center, Beijing, China

**Keywords:** interface-controlled-skin-on-chip, inflammatory skin, drug efficacy, air-liquid interface (ALI), skin barrier

## Abstract

Bacterial skin infections cause a variety of common skin diseases that require drugs that are safer than antibiotics and have fewer side effects. However, for evaluating skin disease drugs, human skin tissue *in vitro* constructed traditionally on Transwell has inefficient screening ability because of its fragile barrier function. With mechanical forces and dynamic flow, the organ-on-a-chip system became an innovative, automatic, and modular way to construct pathological models and analyze effective pharmaceutical ingredients *in vitro*. In this research, we integrated skin extracellular matrix and skin cells into a microfluidic chip to construct a biomimetic “interface-controlled-skin-on-chip” system (IC-SoC), which constructed a stable air–liquid interface (ALI) and necessary mechanical signals for the development of human skin equivalents. The results demonstrated that in the microfluidic system with a flowing microenvironment and ALI, the skin tissue formed *in vitro* could differentiate into more mature tissue morphological structures and improve barrier function. Then, following exposing the skin surface on the IC-SoC to the stimulation of *Propionibacterium acnes* (*P.acnes*) and SLS (sodium lauryl sulfate), the barrier function decreased, as well as inflammatory factors such as IL-1α, IL-8, and PEG2 increased in the medium channel of the IC-SoC. After this pathological skin model was treated with dexamethasone and polyphyllin H, the results showed that polyphyllin H had a significant repair effect on the skin barrier and a significant inhibition effect on the release of inflammation-related cytokines, and the effects were more prominent than dexamethasone. This automated microfluidic system delivers an efficient tissue model for toxicological applications and drug evaluation for bacterial-infected damaged skin instead of animals.

## Introduction

Bacterial infections can cause very common skin diseases that affect life and health (File and Tan, 1991). *Propionibacterium acnes* (*P.acnes*) is a prevalent type of bacteria that colonizes the surface of the skin, which stimulates various types of skin cells to secrete inflammatory cytokines such as tumor necrosis factor (TNF)-α and interleukin (IL) 8 after the microflora is ecologically imbalanced (Kolar et al., 2019; Xu and Li, 2019). Synthetic antibiotics, which have been used to treat various diseases caused by bacteria, may lead to side effects such as pathogen resistance and irritation damage to the skin (Mottin and Suyenaga, 2018; Cong et al., 2019; Xu and Li, 2019; Arpa et al., 2022). Due to the demand for new pharmaceutical ingredients for treating bacterial skin infections, pathological skin models constructed *in vitro* for large-scale screening of effective pharmaceutical ingredients have become a promising avenue for research.

When modeling bacterial infections using 2D cultured cells, because there was no barrier like organ tissue, bacteria existed in the culture medium of cultured cells, which brought the problem of bacterial contamination into the process of cell culture (Campos et al., 2012; Plantamura et al., 2012), making it very difficult to screen for drugs. Now, researchers’ interest in developing models based on 3D organ cells is growing rapidly because, compared with 2D models, these models are closer to the natural human tissue microenvironment and more effective in analyzing human responses to drugs and chemicals (Antoni et al., 2015; Trietsch et al., 2017; Aavani et al., 2022). The typical method of constructing a 3D skin model *in vitro* is to construct a simple skin model, including dermis and epidermis, called SEs (static skin equivalents). Along these lines, many research teams have designed skin models *in vitro* (Kim et al., 2017; Rademacher et al., 2018; Murakami et al., 2021). These constructed SE models have been used to test the chemicals’ skin irritation and evaluate drug components’ permeability and effectiveness (Lee et al., 2014; Kim et al., 2019; Sutterby et al., 2020). Although the traditional skin construction method can build a 3D environment similar to skin tissue *in vivo*, it is still far from the natural skin structure.

In recent years, with the help of organ-on-chip technologies, researchers can design a microenvironment close to physiological conditions to obtain more complete organ structure and mature tissue function *in vitro* (Trietsch et al., 2017; Vunjak-Novakovic et al., 2021). This technology is also gradually applied in skin drug screening and cosmetic efficacy evaluation due to its advantages of building organ barriers, medicinal chemical gradients, and 3D microenvironments, as well as flowing molecular exchange, which seems to be the most suitable platform for testing drug safety and effectiveness before clinical trials (Varga-Medveczky et al., 2021; Cecen et al., 2022; Paggi et al., 2022). An epidermis-on-a-chip was designed based on the development of a novel microengineered system to test the irritation of toxins and non-toxins. Still, the model is limited to assessing the irritation of chemicals on the epidermal layer (Zhang et al., 2021). Varone and Antonio et al. utilized the open-top Chip in the emerging application of organ chips to analyze the efficacy and toxicity of skin-targeted compounds and to study cell–cell interactions in parallel with clinical trials (Varone et al., 2021). However, even though microfluidic chips have made some progress in the construction and research of simulated skin *in vitro*, as previous models did not reflect the complexity of human host–pathogen interaction occurring at the skin surface, a successful application in drug screening is still missing.

In this work, we designed a microfluidic device, interface-controlled-skin-on-chip (IC-SoC), which is based on a polydimethylsiloxane (PDMS) chip with air and liquid channels connected to the culture chamber so that the air–liquid interface (ALI) could be formed in the chamber. The full-thickness skin was constructed and cultured in a culture chamber with a flowing medium separated by a polyethylene terephthalate (PET) film membrane, which could support cell survival and nutrient exchange. The flexible opening above the culture chamber and the air space connecting the airflow channel help improve the construction’s repeatability and further detection. We compared the full-thickness skin in IC-SoC to SEs cultured in standard tissue culture inserts. The results showed that the skin in the IC-SoC showed more mature skin structural differentiation and enhanced dermal–epidermal junction (DEJ) and barrier function, thus reducing skin permeability. Finally, we provided evidence by exposure of the skin’s epidermal side in the IC-SoC to P. acnes and SLS for biological responses to skin infections, and the IC-SoC was utilized to evaluate the therapeutic effects of polyphyllin H (H) and dexamethasone (DEX) on exposure-induced inflammatory skin disease *in vitro*.

## Materials and methods

### Fabrication of the interface-controlled-skin-on-chip mold

The molds were designed using SolidWorks. The platform made of polydimethylsiloxane (PDMS) comprised four parts: a down layer with a bottom microfluidic medium channel which was 1,000 μm (height) × 500 μm (width), a 10 mm diameter bottom chamber, a porous polyethylene terephthalate (PET) membrane (ipCellCulture, it4ip, Belgium) which has a thickness of 50 μm with pores 4 μm diameter and spaced in a 40 μm, up layer with air channel and a culture chamber which has a diameter of 8 mm and a height of 4 mm, and a lid with air and medium inlet and outlet. The lid was connected to the chip using a customized clamping device, which firmly fixed the lid on the top surface of the upper layer of the chip. During the experiment, the lid could be opened and closed reversibly as reagents were added directly or taken out of the skin tissues for further investigation. In addition, the totally transparent lids could help observe the culture process.

PDMS components of different layers were obtained by casting the PDMS mixture (A:B ratio of 9:1) into the mold and then holding it at 80°C for 30 min for solidification. Subsequently, the PDMS is stripped off from the mold, and metal pipes were used to punch chambers in the PDMS chip. The different PDMS components were manually aligned and treated by oxygen plasma (Femto Science Covance oxygen plasma machine) to bond together and cure at 80°C overnight. Before use, sterilize the chip by exposure to ultraviolet light for half an hour.

### Cell culture

Immortalized human HaCaT keratinocytes (ATCC) were maintained under basal conditions using keratinocyte serum-free medium (K-SFM, Thermo Fisher Scientific) supplemented with 0.09 mM calcium, 0.2 ng/ml epidermal growth factor (EGF, Thermo Fisher Scientific), 25 μg/ml bovine pituitary extract (Thermo Fisher Scientific), and 1% penicillin–streptomycin (GIBCO). The medium used in ALI culture is supplemented additionally with 1.2 mM calcium. Dermal fibroblasts (ATCC) were maintained in Dulbecco’s modified Eagle medium (DMEM) supplemented with 10% fetal bovine serum and 1% penicillin–streptomycin (GIBCO). Cultures were incubated at 37 C in a 5% CO_2_ atmosphere, and the culture medium was changed three times a week. Cells of passage numbers 2–9 were used.

### Generation of full-thickness skin

To generate a dermal layer, 100 μL of dermal matrix and eight volumes of collagen I gel solution (10 mg/ml) were mixed with one volume of 10X EMEM (Lonza) and one volume of 10X PBS buffer (Anacker and Moody, 2012) to obtain a collagen solution at a final concentration of 8.0 mg/ml. The proper pH of the collagen mixture was evaluated by the switch from yellow to pink visually; 10 μL of 1 N NaOH was added to 1 ml of collagen mixture to adjust its pH to around 7.4. At this point, one volume of fibroblast solution (2 × 106 cells/ml) was added to the collagen solution and mixed thoroughly. The collagen I hydrogel with embedded fibroblasts was pipetted in the culture chamber of the chip, compressed to 1 mm tall gels, and left polymerized at 37°C with 5% CO_2_ for 60 min.

The SEs (static skin equivalents) were performed in Transwell (Corning, United States). The culture process of full-thickness human SEs was the same as that described in previous studies (Strüver et al., 2017; Mori et al., 2018). Passage 2 HACAT cell suspensions containing 1 × 105 cells were seeded on the top of dermal equivalents in the culture chamber of the device and then cultured in a submerged state until the cells formed a compact monolayer, and then the medium was changed into a high-calcium medium to promote keratinocyte differentiation, stratification, and keratinization.

Also, the culture process of IC-SoC comprises three main phases: the culture of the dermal equivalents, the submerged culture of dermal equivalents and epidermal cells, and the culture at the air–liquid interface (ALI). First, the culture medium was supplied to the device through a sterile syringe filter of 0.22 mm. Then, the medium flows into the upper culture chamber through the medium channel and medium chamber at a flow rate of 1.0 μL min^−1^. Similar to the SE culture, passage 2 HACAT cell suspensions were seeded on top of dermal equivalents. After 2 days, by pumping air at a flow rate of 1 μL min^−1^ in the up-layer air channel for ALI culture, the medium in the medium channel was changed to a high-calcium medium to promote keratinocyte differentiation, stratification, and keratinization.

### Histological and immunofluorescence analysis

The skins of the IC-SoC and the SEs were separated from the chamber by skin biopsy punches and fixed in 10% neutral buffered formalin and then dehydrated by a series of alcohol washes of increasing concentrations (70%, 80%, two of 95%, and three of 100% ethanol) and embedded into paraffin wax. Deparaffinized sections of 5 μm cutting were transferred onto slides for hematoxylin-eosin (HE) staining and immunofluorescence analysis. The HE stained bright-field images were used for the general analysis of morphological structure. The histological characteristics were measured by Caseviewer software. For immunofluorescence staining, the dewaxed and rehydrated specimens were washed in PBS and then blocked in 0.5% bovine serum albumin (BSA) containing 0.025% Triton-X-100 at room temperature for 1 h. Next, they were incubated with primary antibodies, including rabbit anti-keratin-14 (1:500 dilution, Abcam, United States), rabbit anti-loricrin (1:500 dilution, Abcam, United States), and rabbit anti-filaggrin (Abcam), for 1 h. After washing thrice with PBS, the samples were incubated with secondary antibodies: goat anti-rabbit IgG conjugated to Alexa Fluor 488 (1:1,000 dilution, Invitrogen, United States) for 1 h at RT. Finally, the slides were stained with 4′,6-diamidino-2- phenylindole (DAPI) (Thermo Fisher Scientific, United States) for 3 min, and the fluorescence images were observed and photographed by fluorescence microscope (Olympus, Japan) and analyzed by ImageJ.

For the image of basement membrane (BM) proteins at the dermal–epidermal junction (DEJ), the sections were incubated in type IV collagen (1:200 dilution, ABCAM, United States) for 1.5 h and then incubated in HRP-conjugated secondary antibody at a dilution of 1:100 for 30 min and then lightly stained sections with hematoxylin. The primary antibody was the negative control. For checking the mitosis of the keratinocytes, Ki67 was stained with antibody (1:50 dilution, ABCAM ab281928 red, United States) for 1 h. After washing thrice with PBS, images were observed and photographed by a fluorescence microscope (Olympus, Japan) and analyzed by ImageJ.

### Transepidermal electrical resistance (TEER) measurement and skin permeation

EVOM2 (World Precision Instruments, United States) was used to measure the skin resistance in IC-SoC and SEs at different ALI culture times to study the skin barrier function. Four pieces of platinum (Pt) electrodes were inserted into the device, whereas two electrodes were inserted into the upper culture chamber and immersed with PBS added to the skin surface. The other two electrodes were inserted into the outlet of the down medium chamber. The resistance containing medium and membrane without a cell device was used as a blank control. To avoid temperature interference, the medium and PBS used should be placed at room temperature for at least 30 min. The measured TEER value of the sample subtracts the value of the blank control group (the TEER values of Transwell or IC-SoC without cells), and then the resistance value per unit skin area is obtained according to the size of the sample area.

To test the permeability of the skin sample, 70 kDa dextran-FITC solution (10 μM) was added to the surface of the sample and tested every 2 h. The steps are as follows: 50 μL of PBS was pre-placed in the medium chamber below the chip or dish, and then 10 μL of FITC test solution was added to the top of the sample and incubated at 37°C. At the detection point, 50 μL solution is transferred from the medium chamber at the bottom of the insert or the medium outlet of IC-SoC to the 96-well plate. Then, the microplate reader (Bio-tek, United States) was used to measure the cascade green value at the excitation wavelength of 488 nm and emission wavelength of 520 nm to obtain the amount of dextran to characterize the permeability of skin samples.

### Chemicals and drug treatment and inflammatory cytokine detection

In brief, several groups of IC-SoC of ALI culture were prepared. Then, for the skin infection model, 5 μL of 0.2% SLS and 5 μL of 108 CFU/ml *Propionibacterium acnes* were added and washed off with 0.05 ml of PBS 3 times after the designed hours of contact. After that, the dosing solutions of each drug sample were proved. Finally, the chips were incubated at 37 C with 5% CO_2_ and recirculated in the medium channel with a culture medium.

After the skin tissue in the upper culture chamber of IC-SoC was treated with designed irritants and drugs, the chip was incubated with 5% CO_2_ in a humidified atmosphere at 37°C. The effluent of the medium channel was collected at a set time and diluted. The ELISA kits (Proteintech, United States) were used to detect cytokines (PEG2, IL-8, and IL-1α). The detection process did not disassemble the chip culture chamber, and the tissue could be retained for further characterization.

### Tissue cellular viability

3-(4,5-dimethylthiazol-2-yl)-2,5 diphenyl tetrazolium bromide (MTT, Sigma-Aldrich) was used to assess tissue cell viability. The steps are as follows: wash the sample three times in PBS, add 50% 50 μL MTT solution (0.5 mg ml−1 MTT) to each tissue chamber, and then incubate in the incubator for 5 h. Then, 1 ml of iso-propanol was circulated through the down layer culture medium channel for 2 h to obtain the purple-colored formazan salt released from live cells. A microplate reader (Bio-tek, United States) was used to measure the optical absorbance at 540 nm. Set PBS as the negative control and 5% SDS as the positive control. Then, calculate the percentage of cell viability (C) value of the sample using the following equation: C%= ((As-Ap)/(An-Ap))*100, where As, An, and Ap correspond to the value of the sample, negative control, and positive control, respectively. All the tests were taken at five repetitions.

### Statistical analysis

Five repeated skin structures were designed for the detection of barrier function and cytokines in each group. The data obtained were statistically analyzed by the software GraphPad Prism using one-way ANOVA or Student’s t-test with a 95% confidence interval. The data are shown as means ± SEM. *p* < 0.05 was considered a significant difference.

## Results

### Design and modeling of integrated interface-controlled-skin-on-chip

**FIGURE 1:**
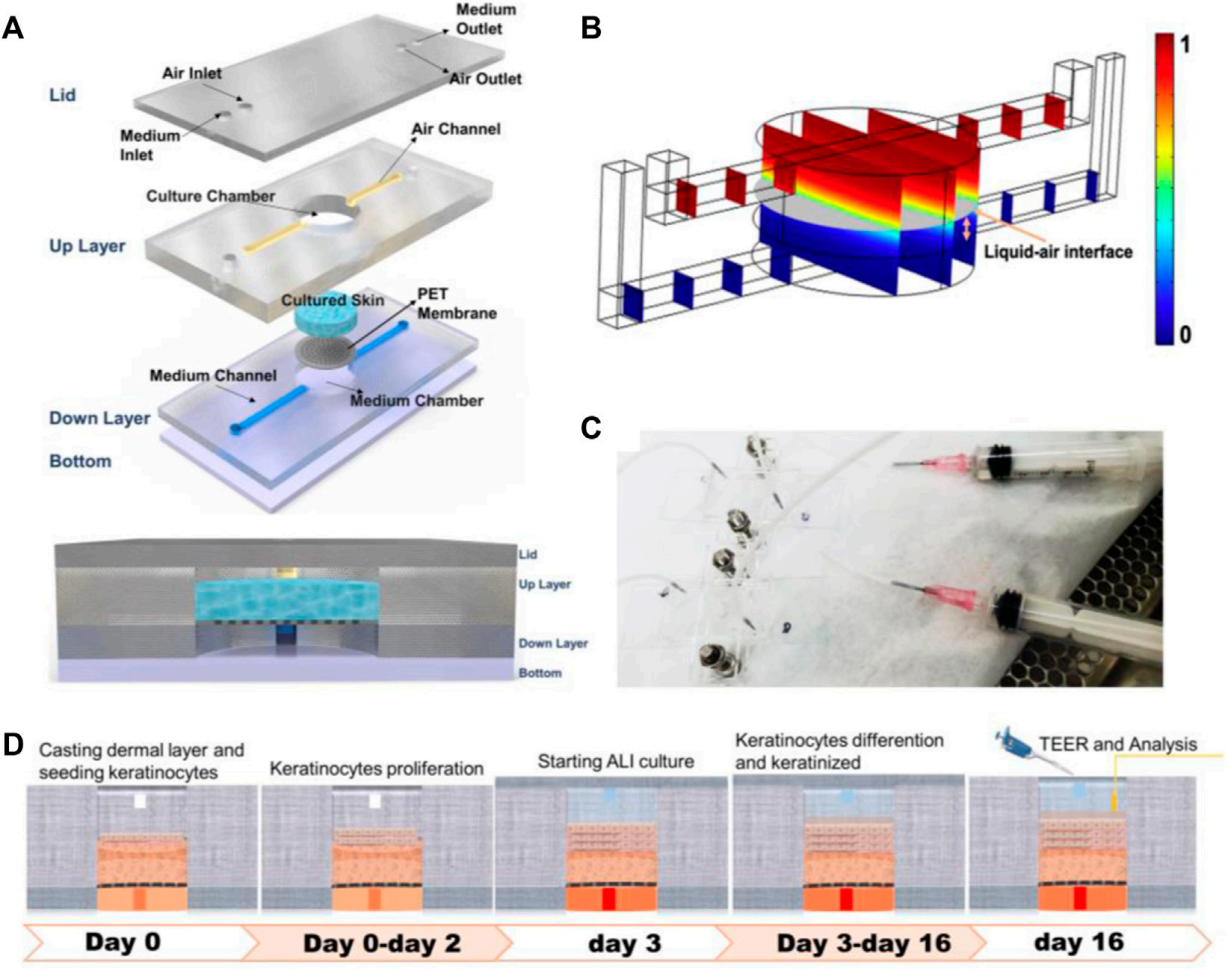
Design and simulation of IC-SoC. **(A)** Exploded view of the IC-SoC device composed of four major layers and a membrane. **(B)** COMSOL Multiphysics simulation of air-liquid distribution in the microchannels. The gray plane represents the air–liquid interface, and the color bar shows the volume fraction of air. **(C)** Appearance of IC-SoC connected with the fluid injector. **(D)** Schematic overview of the full-thickness skin culture on chip from IC-SoC cross section and *in vitro* assay protocols.

This IC-SoC (interface-controlled-skin-on-chip) (Figure 1A) was composed of a culture unit and flow connection. Sandwiched between the bottom chamber with microfluidic channels and the circular tissue culture chamber was the flexible porous PET membrane, which provided a supporting structure for tissue culture and a diffusion interface for culture medium, metabolites, or test components. The IC-SoC was connected with a liquid syringe at the inlet of the culture medium and injects the culture medium into the culture medium chamber at the down layer, supplies the culture medium, and then circulates through the flow channel at the down layer, so as to flow out of the culture medium outlet (Figure 1C).

The designed microdevice was appropriate for ALI (air–liquid interface) culturing. Usually, the location of the air–liquid interface in the microfluidic chamber could be controlled by means of wettability regulation, microfluidic structure design (Li et al., 2022), air-liquid flow rate ratio regulation, etc (Huang et al., 2006; Oliveira et al., 2019). In this case, we intuitively observed the air–liquid interface heights in our microfluidic chamber at different air-liquid flow rate ratios through COMSOL Multiphysics simulation (Figure 1B). According to the need to form 1 mm thick skin, the thickness of the skin constructed by this method would be within the thickness range of the epidermis plus dermis of the normal human face, and the microfluidic conditions suitable for skin tissue culture on IC-SoC were further determined. This microfluidic platform is especially suitable for barrier tissue because of the nutrient microenvironment with continuous gradient and the shear stress generated by fluid flow. The distribution of the interfacial of the cultured tissue at a specific flow rate and the highest regional shear stress reached 3 × 10^−4^ dyn/cm, which we used in the chip would not harm the cells.

### Long-term differentiation of skin in interface-controlled-skin-on-chip

**FIGURE 2:**
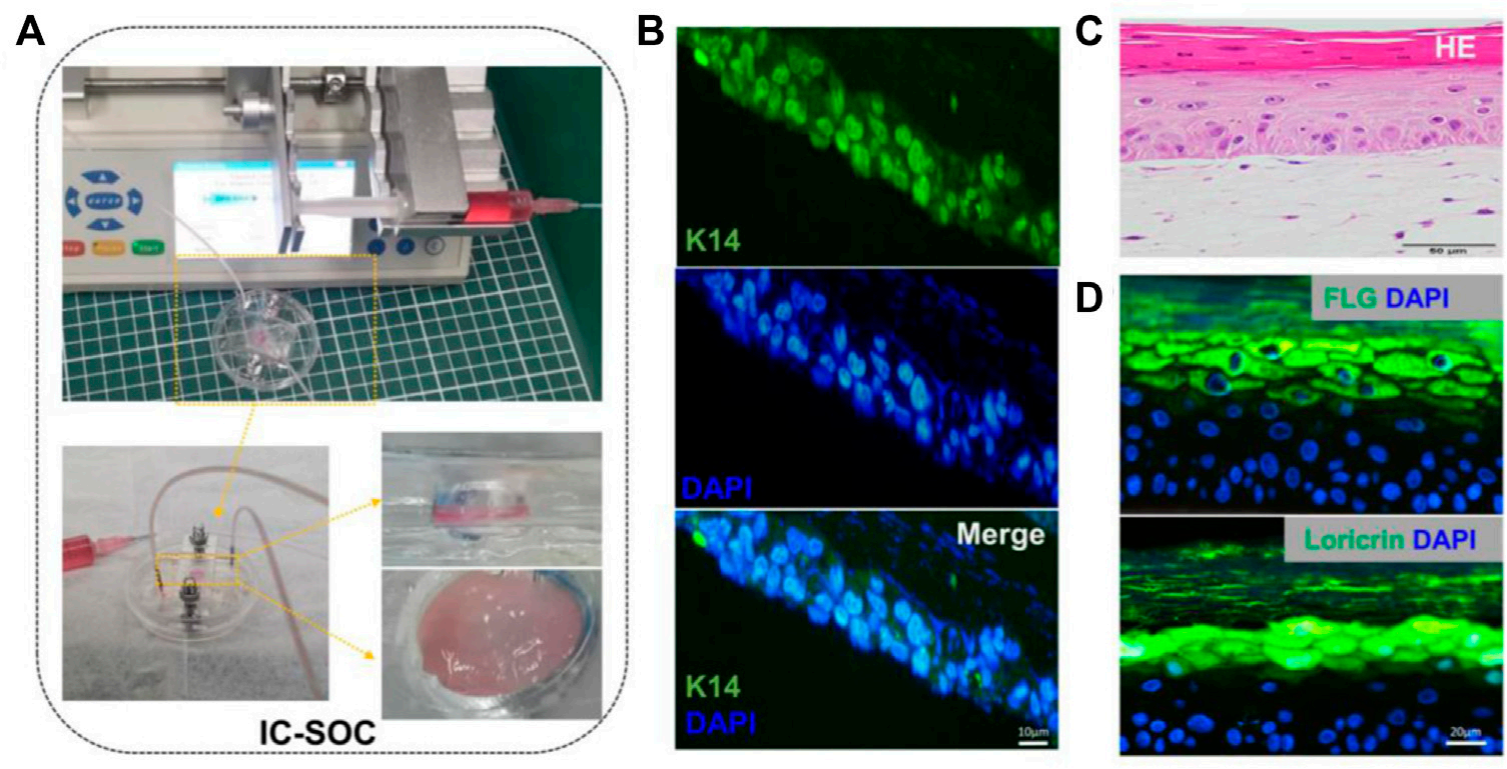
Morphology and cell characterization of IC-SoC. **(A)** Upper part shows the skin cultured in IC-SoC, the lower part is the enlarged skin in the culture chamber, and the right part shows the side view and the top view of the skin. **(B)** IC-SoC reveals expression of K14 (a marker for the initiation of keratinocyte differentiation) after 7 days of air–liquid culture. Scale bars: 10 μm. **(C)** H&E cross section of the skin epidermis in the IC-SoC. **(D)** Expression of differentiation markers FLG (filaggrin) and loricrin after 14 days of ALI culture. Nuclei are stained with DAPI (blue). Scale bars: 20 μm.

After completing the dermal construction with collagen scaffolds and fibroblasts, keratinocytes were added to the dermis to cover the surface and exposed to air. Then, the construction matured at ALI, keratinocytes were stratified, and keratinized epidermal differentiation was completed (Figure 1D). The results of this culture process show that a 3D skin tissue structure with a specific thickness was formed in the culture chamber of IC-SoC (Figure 2A). Generally, the expression of keratin-14 (K14) near the basal layer of epidermis indicates that keratinocytes establish appropriate localization and enter the stage of differentiation and keratinization. (Figure 2B). After 2 weeks of culture at ALI, the full-thickness human skins of IC-SoC were evaluated, and the histological features of the sample demonstrated the keratinocytes in the chip could form a mature epidermis with more than five cell layers on the dermis constructed of fibroblasts and collagen (Figure 2C). The epidermis is characterized by the formation of the basal, spinous, granular, and stratum corneum, which together represent normal human skin (Egawa and Kabashima, 2018). Similar to normal human skin, the IC-CoC demonstrated filaggrin (FLG) and loricrin expression (Figure 2D). FLG mainly exists in the granular layer and keratinized stratum corneum, and the late differentiation marker loricrin is distributed in the granular layer (Kim and Lim, 2021). Their expression indicates the existence of a granular layer and keratinized stratum corneum and the differentiation hallmark of epidermal maturation. In conclusion, these characteristics showed that mature skin structure and complete morphology could be formed under the culture conditions of IC-SoC.

### The barrier function of the skin enhanced in the IC-SoC culture

**FIGURE 3:**
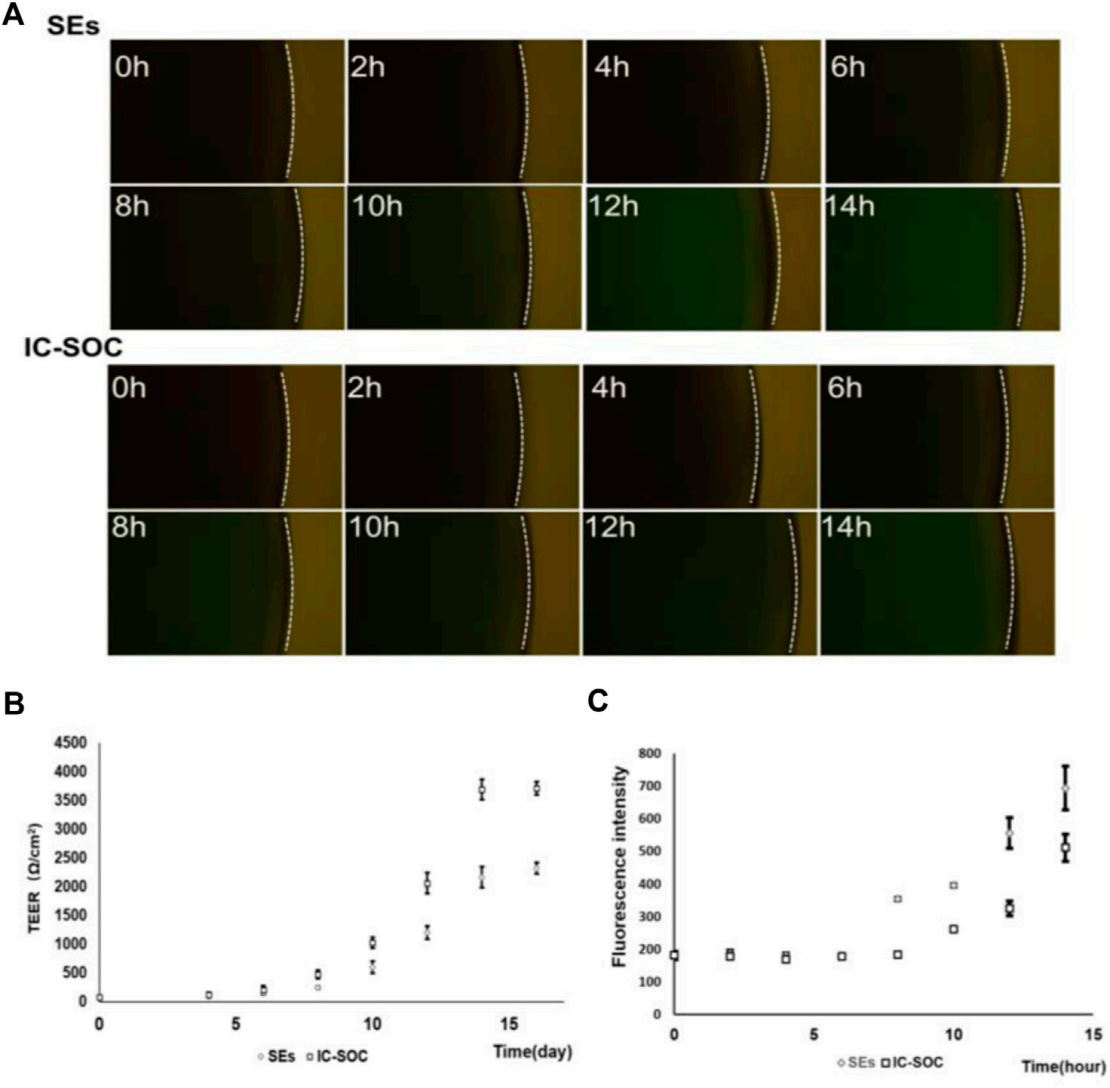
Barrier function analysis of the IC-SoC. **(A)** Fluorescent molecule permeability of the SEs and the IC-SoC. **(B)** TEER values of the IC-SoC within 14 days of air–liquid culture. **(C)** Values of cascade green as the SEs and the IC-SoC **(A)**, which showed the fluorescence intensity of molecules penetrated after different hours (*n* = 5 per condition).

TEER is a rapid technology without labeling to study skin integrity. TEER is a non-invasive technology suitable for continuously monitoring barrier function (Zhang et al., 2021). The SEs (static skin equivalents) were performed in the stationary Transwell method (Supplementary Figure S1A). The barrier function of SEs was characterized by comparing the difference in TEER values between SEs and monolayer cells. The mean resistance of SE skin samples was 2.6 ± 0.3 kΩ/cm2, which was significantly larger than that of the cell layer (Supplementary Figure S1B). In the monitoring of IC-SoC, continuous TEER values showed a gradual increase in process with the increase of culture time (Figure 3B). Along with ALI culture, TEER values of the IC-SoC were gradually higher than those of SEs (Figure 3B), and the steady increase of TEER values was the manifestation of the enhancement of skin barrier function in the IC-SoC.

Additionally, in order to study the skin barrier function and the skin permeability of specific molecules, the solution containing the 70 kDa dextran-FITC was added to the skin surface of IC-SoC for experimental testing. The barrier function of the skin and the permeability to 70 kD dextran were measured at specific time intervals by collecting dextran-FITC from the medium flow channel and reading the fluorescence signal intensity. The results in Figure 3A revealed that the skin in IC-SoC could significantly prevent a molecule like cascade 70 kDa compared to the SEs. Obviously, the permeability of IC-SoC is lower than that of SEs, with a difference of more than 1.5 times (Figure 3C). The low permeability of molecules on the skin is correlated with the high TEER value in IC-SoC, which indicates that the skin of IC-SoC had a stronger barrier function and sufficient resistance to substances outside the skin.

### Microfluidics favors the synthesis of basement membrane proteins

**FIGURE 4:**
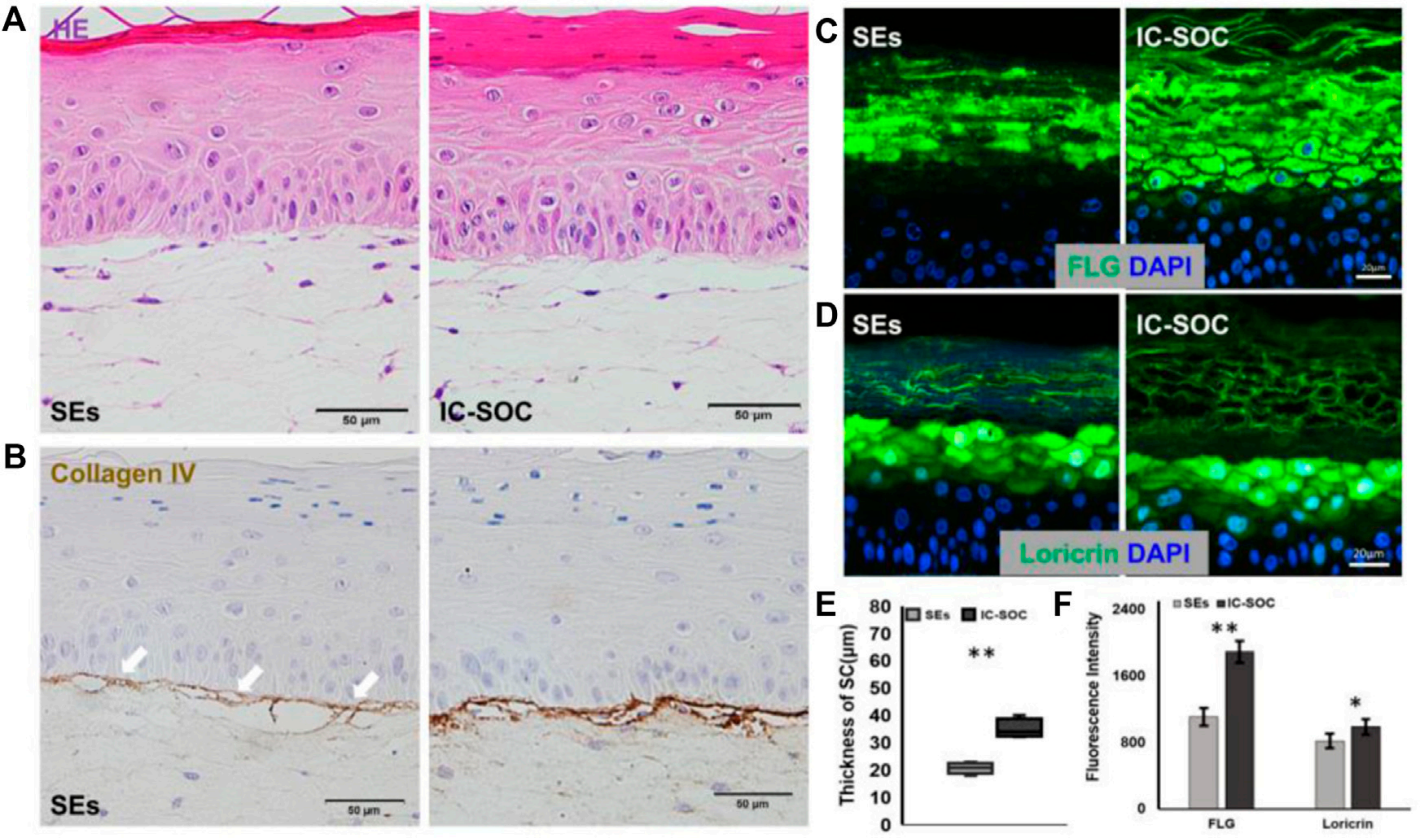
Impact of microfluidics on basement membrane development. **(A)** H&E cross section of the skin in the SEs and IC-SoC. **(B)** Basement membrane as revealed by collagen type IV expression, which is indicated by white arrows. Scale bars: 50 μm. **(C,D)** Expression of differentiation markers FLG: filaggrin **(C)** and loricrin **(D)** in epidermis of the SEs and IC-SoC. **(E)** Statistical analysis of SC thicknesses in the skin of the SEs and IC-SoC. **(F)** Statistical analysis of expression of FLG and loricrin (*n* = 5 per condition, p**<0.01; p***<0.001).

In order to explore the reasons for the difference in skin barrier function between the two models, we compared the tissue structure of the skin and the expression of specific proteins. The histological features showed that the skin cultured in IC-SoC had a thicker stratum corneum structure than SEs (Figure 4A), and the mean thickness was 1.5-fold greater (Figure 4E). Type IV collagen, as the most important expression component of the basement membrane (BM), could anchor the epidermis to the dermal tissue, which is an important feature of dermal–epidermal junction (DEJ) structural integrity (Vázquez et al., 1996; Behrens et al., 2012). Compared with the skin in the chip, the expression of type IV collagen in SEs was less, and there were more morphological fractures between the epidermis and dermis even after an extended culture period (Figure 4B). This result was consistent with the poor barrier function of SEs. In contrast, the expression of type IV collagen deposited in the BM region of the skin DEJ structure cultured in the chip was more abundant (Figure 4B). By immunofluorescence staining, it was observed that the expression of FLG (Figure 4C) and loricrin (Figure 4D) in the stratum corneum of the IC-SoC epidermis was significantly more than that of SEs (Figure 4F). Therefore, skin cultured in IC-SoC had a thicker stratum corneum and enhanced DEJ structure than reconstructed SEs, which further explained its better barrier function under dynamic culture conditions.

### Evaluation of the irritation and infection response in skin

**FIGURE 5:**
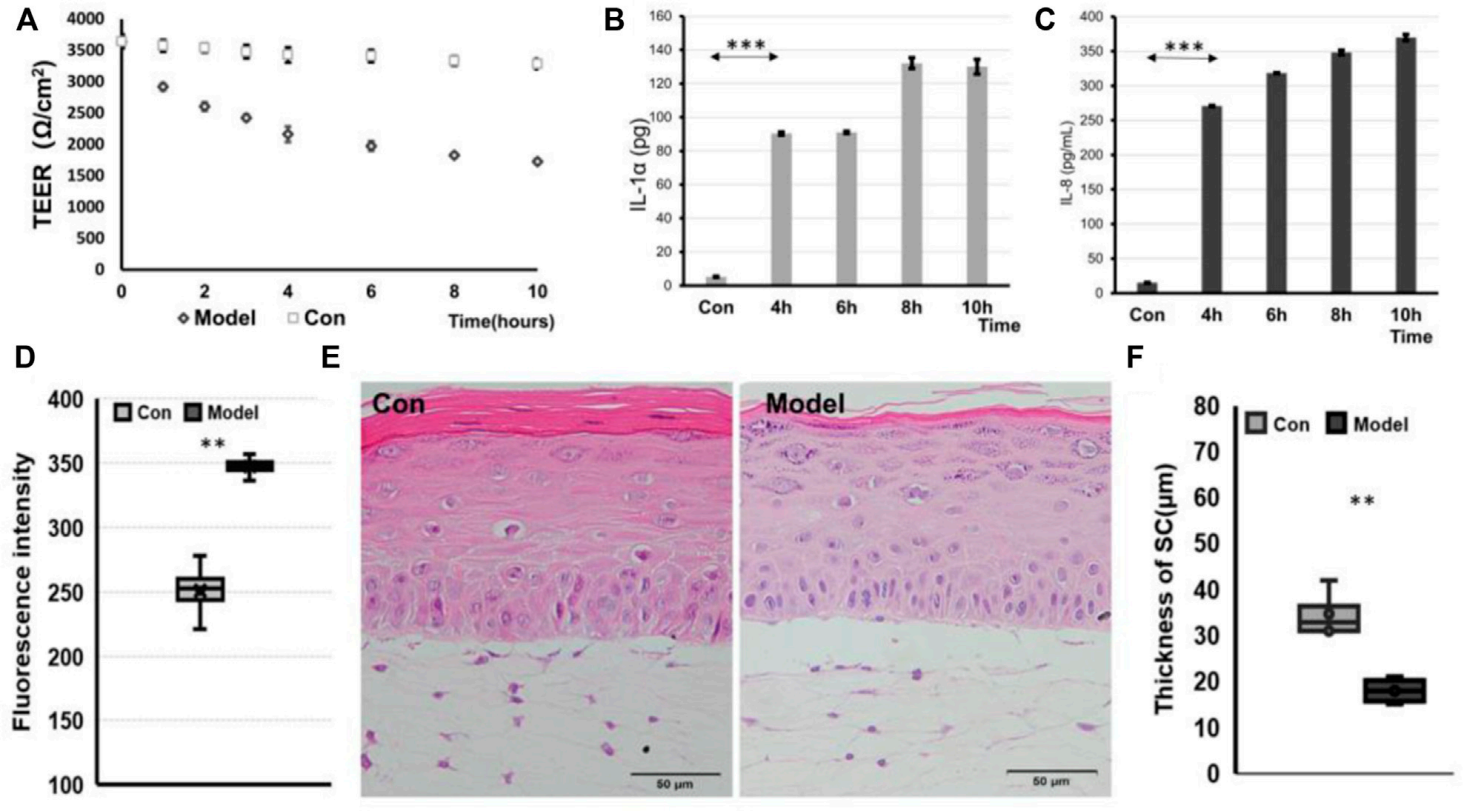
IC-SoC inflammatory response. **(A)** TEER values of control (Con) and model (exposure to SLS + *P.acnes*) at different times in response to stimulation. **(B)** and **(C)** graphs showing the release of IL-1α and IL-8 cytokines in response to stimulation. **(D)** Fluorescent molecule permeability of control (Con) and model (exposure to SLS + *P.acnes*) for 10 h. **(E)** H&E cross section of control (Con and model. **(F)** Statistical analysis of SC thicknesses in the skin of control (Con) and model (*n* = 5 per condition, p**<0.01; p***<0.001).

As an alternative to animal experiments, tissue-engineered skin models have been used in testing, research, and analysis, especially in the skin irritation of chemical components, the safety of skin medication, and the efficacy of cosmetics. To evaluate whether this model enables the triggering of an inflammatory response as a skin acne inflammation model, in our study, we used the stimulation methods used by other researchers, which can stimulate skin cells to initiate an inflammatory response (Barba et al., 2019). After treating the skin of IC-SoC separately with only SLS or *P. acnes* for 28 h, the TEER values of the skin declined by 11% in *P. acnes* and 29% in SLS (Supplementary Figure S2). But following irritation of the skin of IC-SoC with SLS and *P. acnes* for 10 h, the TEER values of the skin declined 44% significantly (Figure 5A). Acne usually occurs when both barriers are damaged and accompanied by a bacterial infection (Mottin and Suyenaga, 2018; Xu and Li, 2019). For the sake of experimental efficiency and a more realistic simulation of acne, irritation with SLS plus *P. acnes* was carried out. The permeability of these IC-SoC was detected to increase after irritation for 10 h (Figure 5D). For inflammatory response, upregulation of the inflammatory mediators PEG2, IL-1α, and IL-8 detected in the medium channel of the chip was very significant from 4 h after stimulation (Figures 5B, C, and Supplementary Figure S4). According to the morphological observation of skin histological sections, the stratum corneum layer of the stimulated skin epidermis was damaged, and the connection between the skin epidermis and dermis was broken after stimulation for 10 h (Figure 5E). Also, after irritation, FLG immunofluorescence was significantly less than that in the control group (Supplementary Figure S3), and the thickness of the stratum corneum decreased by 48% (Figure 5F). These results suggest that after chemical exposure and bacterial infection, the skin had structural damage, reduced barrier function, and an inflammatory response.

### Analysis of drug efficacy

**FIGURE 6:**
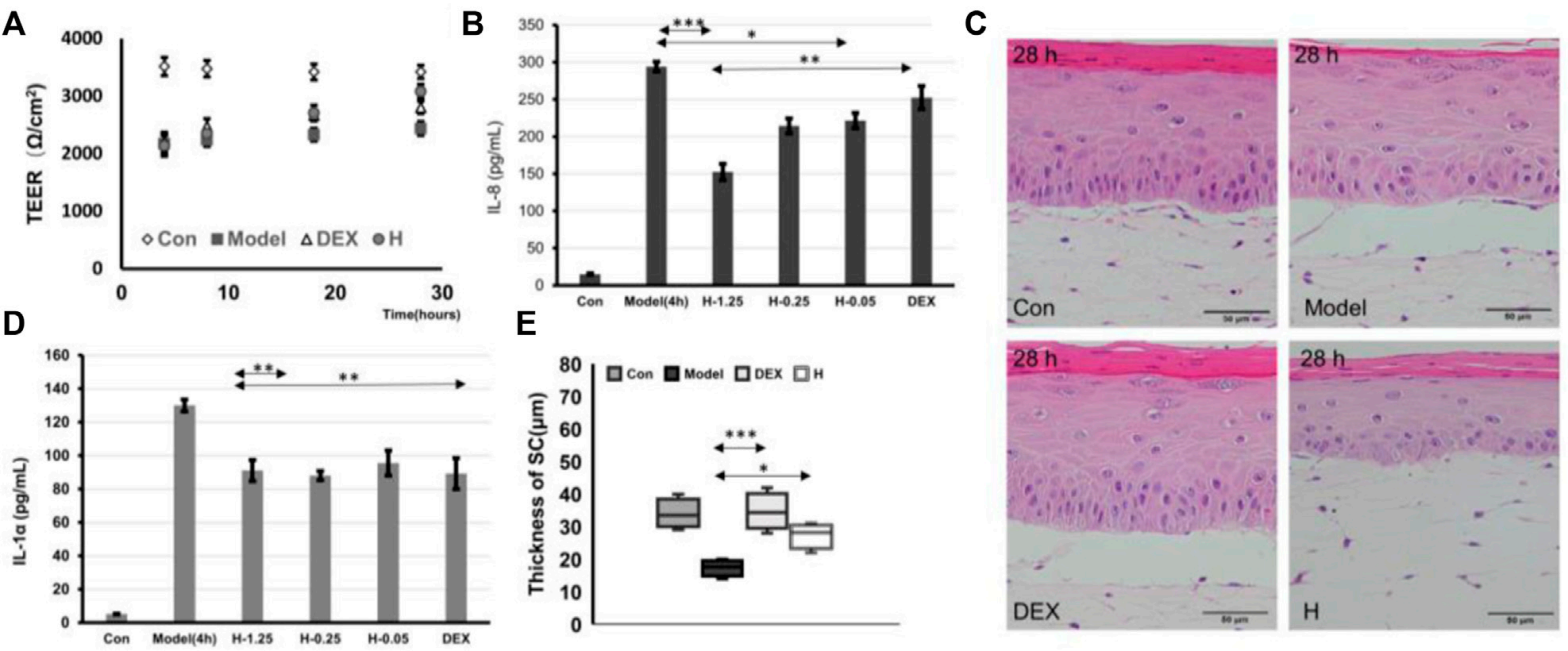
Drug testing. **(A)** TEER values of Con (treated with PBS) and model (treated with PBS for 24 h after SLS + *P.acnes* stimulation for 4 h), DEX (treated with dexamethasone for 24 h after SLS + *P.acnes* stimulation for 4 h), and H (treated with 1.25 μM polyphyllin H for 24 h after SLS + *P.acnes* stimulation for 4 h). **(B,D)** Graphs showing the release of IL-1α and IL-8 cytokines in response to stimulation in the model group and DEX group (treated with dexamethasone for 12 h after SLS + *P.acnes* stimulation for 4 h) and H-1.25, H-0.25, and H-0.05 (treated with 1.25, 0.25, 0.05 μM of polyphyllin H for 12 h after SLS + *P.acnes* stimulation for 4 h). **(C,E)** H&E cross section **(C)** and the thickness of the stratum corneum **(E)** of Con, model, + DEX, and + H in **(A)** (*n* = 5 per condition, p*<0.05; p**<0.01; p***<0.001).

To further demonstrate the possibility that the IC-SoC can be used to evaluate drug efficacy, skin inflammatory pathological drugs, polyphyllin (Wang et al., 2018) and dexamethasone (Tu et al., 2020), which had an inhibitory effect on inflammatory factors in previous studies, were used to test irritation-relevant responses in IC-SoC. First, tissue cellular viability was detected as shown in Supplementary Figure S5A. In the cell model, after treatment, the inhibitory regulatory effect of two drugs on the inflammatory factor IL-8 was found (Supplementary Figure S5B). At the same time, on the pathological inflammation model of IC-SoC, we tested the barrier effect of the two drug components. After comparing the treatment of drugs in IC-SoC, we found that both polyphyllin H and dexamethasone could promote the recovery of the damaged skin barrier, and dexamethasone could recover the skin barrier faster (Figure 6A). However, after 24 h, the skin barrier resistance of the experimental group treated with polyphyllin H was higher (Figure 6A), indicating that the recovery was better. Also, immunostaining against Ki67 showed that the expression of Ki67 was mainly distributed in the basal layer and the upper layer of the basement in the control group. The expression of Ki67 in the model group was higher than that in the control group, but the expression of Ki67 treated with polyphyllin H was more than that in the model group after treatment for 24 h (Supplementary Figure S6), indicating that polyphyllin H promoted the mitosis of keratinocytes. As shown in Figures 6B and D, the *P. acnes*-stimulated inflammatory cytokine secretion was significantly suppressed by polyphyllin H and dexamethasone in the skin model of pathological inflammation. With the increase in the concentration of polyphyllin H, the inhibition rate of cytokines was more obvious. A high concentration of polyphyllin H could alleviate the production of inflammatory factors, and the effect was more prominent than dexamethasone. As analyzing the restoration of skin structure from the morphological features after treatment with the two drugs, the thickness of the stratum corneum after two drug treatments increased significantly (Figure 6E), and the skin tissue on the chip treated with polyphyllin H had a more complete structure in the tissue section (Figure 6C), which was consistent with the TEER value results.

## Discussion

At present, the *in vitro* animal substitution model widely used in basic skin biology research, skin toxicology, and pharmacology is the SE (static skin equivalent) model. However, due to the fragile dermal–epidermal connection structure, incomplete barrier function, and thin stratum corneum, there are still many problems in the research (Kim et al., 2018). The DEJ (dermal–epidermal junction) of intact skin consists of a specific complex component of specialized extracellular matrix, which provides the anchoring of epidermal structure and dermis, and defects of BM (basement membrane) proteins in this complex DEJ assembly result in skin tissue fragility disorders (Aleemardani et al., 2021). Using a microfluidic environment, we reconstructed a mature full skin in IC-SoC (interface-controlled-skin-on-chip) with pluristratified epidermis, more robust DEJ with more abundant collagen type IV expression, and enhanced skin barrier functions. Hyperplasia, hyperkeratosis, and parakeratosis often occur in the skin of patients with psoriasis, while the stratum corneum is destroyed and thinned, resulting in a decrease in barrier function (Zingkou et al., 2022). The thickness of the nuclei-free stratum corneum in IC-SoC was thicker than the static-cultured SEs (Figure 4E), which also might be one of the reasons for affecting barrier function. Some skin samples in IC-SoC had observed the simultaneous presence of cells with FLG and nucleus, mainly at the junction of the stratum corneum and the granular layer, which might be caused by insufficient keratinization time in the culture process, and the relationship between nuclear appearance and transepidermal barrier function deserves more research.

The pulsatile nature of the peristaltic flow may induce mechanical stretching on the support membrane and consequently on the skin (Sriram et al., 2018). On the one hand, IC-SoC provides continuous nutrient supply and skin tissue metabolite circulation by the medium channel, which is similar to the role of the capillaries in skin tissue, and ALI (air–liquid interface) is similar to the microenvironment of skin tissue that promotes the maturation of epidermal and barrier functions. The medium flowing from the medium chamber produced interstitial flow and chemical pressure gradient in the culture chamber tissue of IC-SoC, which might increase the signal interaction between the dermal matrix and the epidermal layer during ALI culture, which was different from the static SE culture in which nutrient transport was purely the diffusion from medium to the dermal matrix. Also, the dermal matrix scaffold used to construct the skin on the chip might generate a mechanotransduction mechanism, whose effects were transmitted to the deeper epidermal layers, thus promoting a stronger anchoring of the basal layer to the dermal matrix (Kim et al., 2019). On the other hand, the IC-SoC maintained the capability for operating and analyzing the skin of the culture chamber and the channel containing the culture medium independently. Secreted products collected from the medium channels could be collected at a specific time, which allowed accurate characterization of dynamic secretions and metabolites. Also, with the microfluidic system design, IC-SoC could complete the TEER value measurement without disassembling the chip to move the skin tissue out, so that it could maintain the continuous culture process. This efficient chip could reduce the manual operation of collecting a large number of experimental samples so as to reduce personal errors and help improve the repeatability of experiments.

Most serious skin inflammatory diseases are caused by various microbial infections, among which *Propionibacterium acnes* are also the main cause of inflammatory skin diseases such as skin acne, especially when the skin is damaged (Bay and Ring, 2021). Previous studies have shown that both SLS (Barba et al., 2019) and *P. acnes* (Xu and Li, 2019) could stimulate the skin and damage the barrier. With the advantages of IC-SoC, the pathological model of skin inflammation caused by the co-stimulation of SLS and *Propionibacterium acnes* was constructed. FLG and the thickness of the stratum corneum were significantly reduced after irritation with SLS and *P. acnes*, which provided evidence of damage to the stratum corneum concerning the barrier function. This result might be due to the aggravation of bacterial proliferation and infection after SLS destroys the stratum corneum structure. IL-8 is perhaps best known for its proinflammatory effects on immune cells. A wide variety of cells secrete IL-8, and these include fibroblasts and keratinocytes (Lim et al., 2009). As in previous studies, the production of IL-6 and IL-8 could lead to the depletion of hemidesmosomes and inflammatory factors could directly deplete BP180, thereby leading to fragility of the dermal–epidermal junction (Iwata et al., 2009; Liu et al., 2017). In our study, an increase in inflammatory factors after stimulation and the destruction of the stratum corneum and the dermal–epidermal junction were also observed, and these results were consistent with previous studies.

Steroids and antibiotics are the main drug components for the treatment of skin infections at present, but the long-term use of these has different side effects (Eichenfield et al., 2021). Therefore, more drug components for the treatment of skin inflammatory diseases are needed, especially those from natural plants, which also attract the attention of researchers (Mohd Zaid et al., 2022). This study also explored the effect of polyphyllin H on inflammatory skin diseases induced by *Propionibacterium acnes in vitro*. Using this pathological model, we found that both polyphyllin H and dexamethasone could significantly reduce the production of the pro-inflammatory cytokines. Also, the results indicated that polyphyllin H from natural plants could repair the skin damage barrier and regulate the secretion of inflammatory factors better than dexamethasone.

Finally, with the advances in stem cells, sebaceous gland cells, and melanocytes (Liu et al., 2019; Çankirili et al., 2020), the cells contained in the skin chip will be closer not only to keratinocytes and fibroblasts used here but also to human natural skin. Collagen was used in the dermis of IC-SoC, and the difference between the stiffness of the 3D skin model and the natural skin also needs further study. With the advancement of hydrogels as 3D scaffolds (Ding et al., 2020; Montero et al., 2021) and 3D printing (Diogo et al., 2020) for tissue engineering, modeling the stiffness of human skin may help accelerate the development of personalized skin models.

## Conclusion

In this study, we developed a full-thickness skin model in an IC-SoC (interface-controlled-skin-on-chip) system with a more complete structure and enhanced barrier function. This chip was utilized to construct a skin infection and injury disease model and to evaluate and analyze two different anti-inflammatory drug components. The organ-on-chip device is a versatile platform for the reconstruction of epithelial tissues; in the future, this model can also be further upgraded with the design of a multi-culture chamber and become a platform for high-throughput drug screening and analysis.

## Data Availability

The raw data supporting the conclusion of this article will be made available by the authors, without undue reservation.
