# Supplementary material for: Analysis of drug efficacy for inflammatory skin on an organ-chip system
Source: Front Bioeng Biotechnol. 2022 Sep 2;10:939629. doi: 10.3389/fbioe.2022.939629 (PMC9478476; doi:10.3389/fbioe.2022.939629)
Supplement: Supplementary file 1 [file DataSheet1.docx]

**Analysis of drug efficacy for inflammatory skin on an organ-chip system**

**Supplementary**


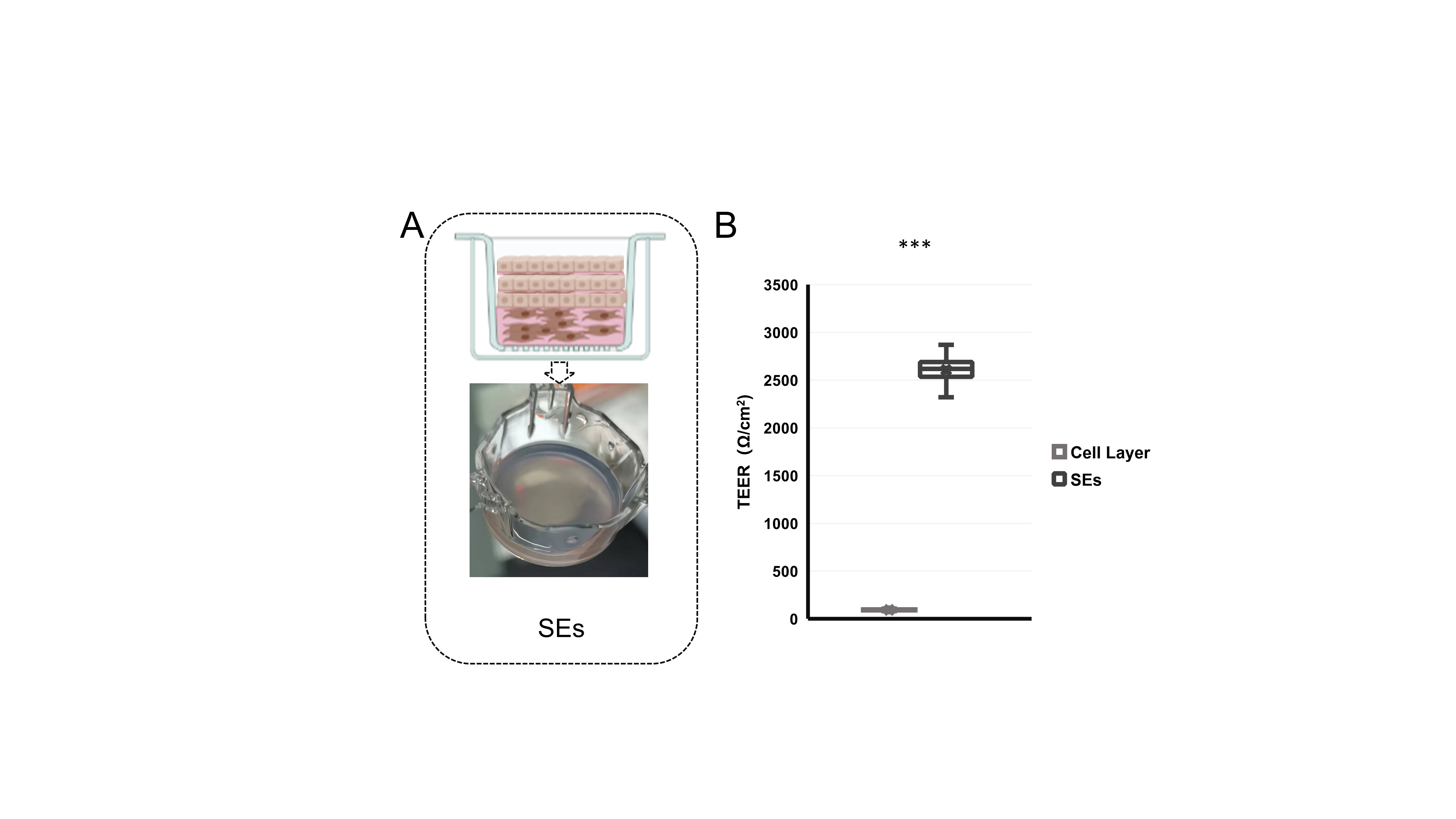


Fig.S1 Characterization of SEs (static skin equivalents). (A) Traditional culturing of SEs. (B) TEER values of cell layer and SEs.


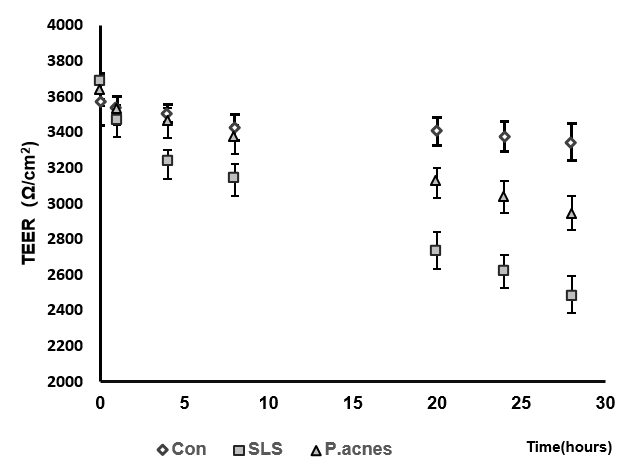


Fig.S2 TEER values of response to stimulation at different times during treating the skin of IC-SoC separately with only SLS or *P.acnes*.


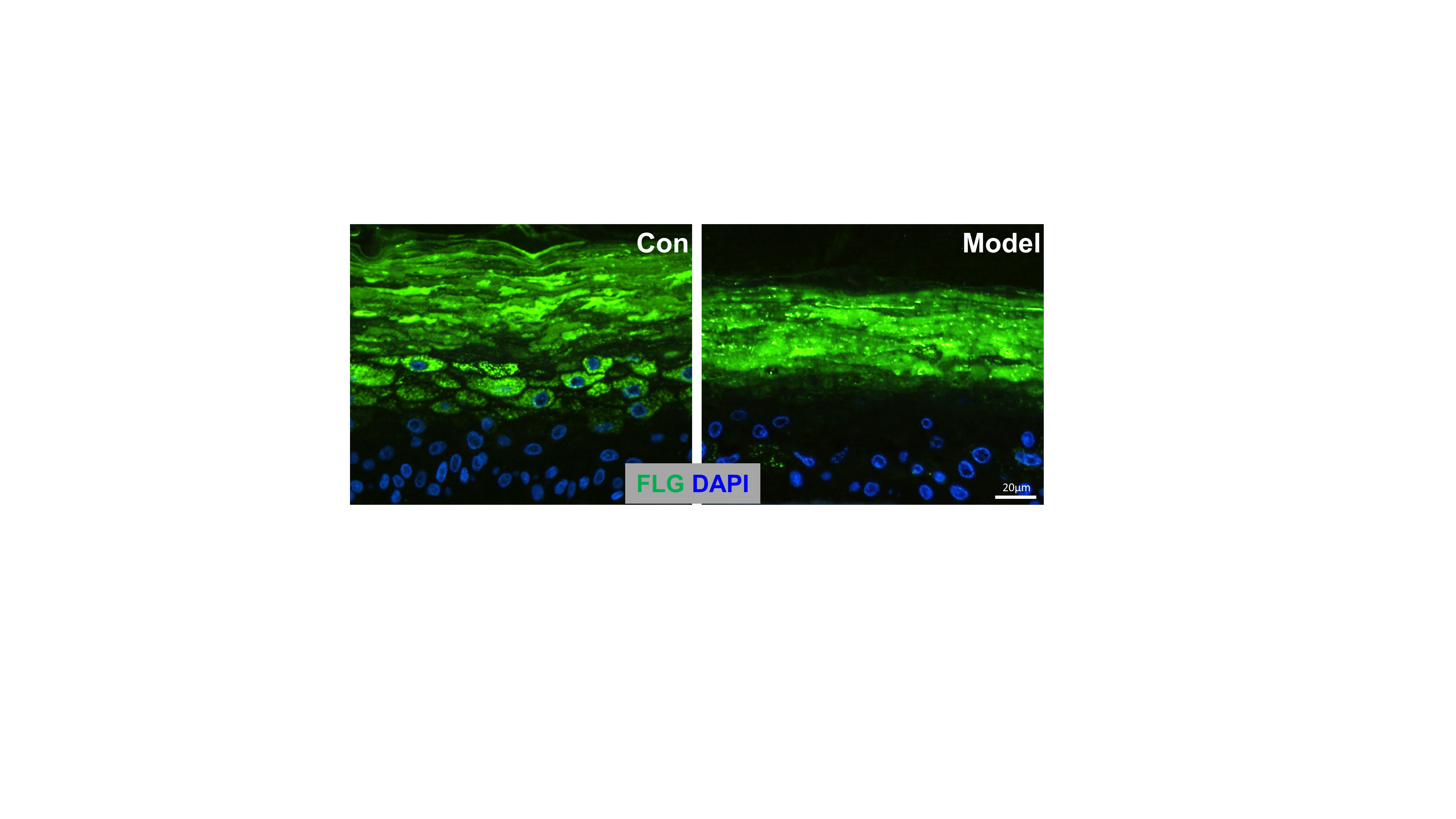


Fig.S3 FLG in skin epidermis of control (Con) and Model.


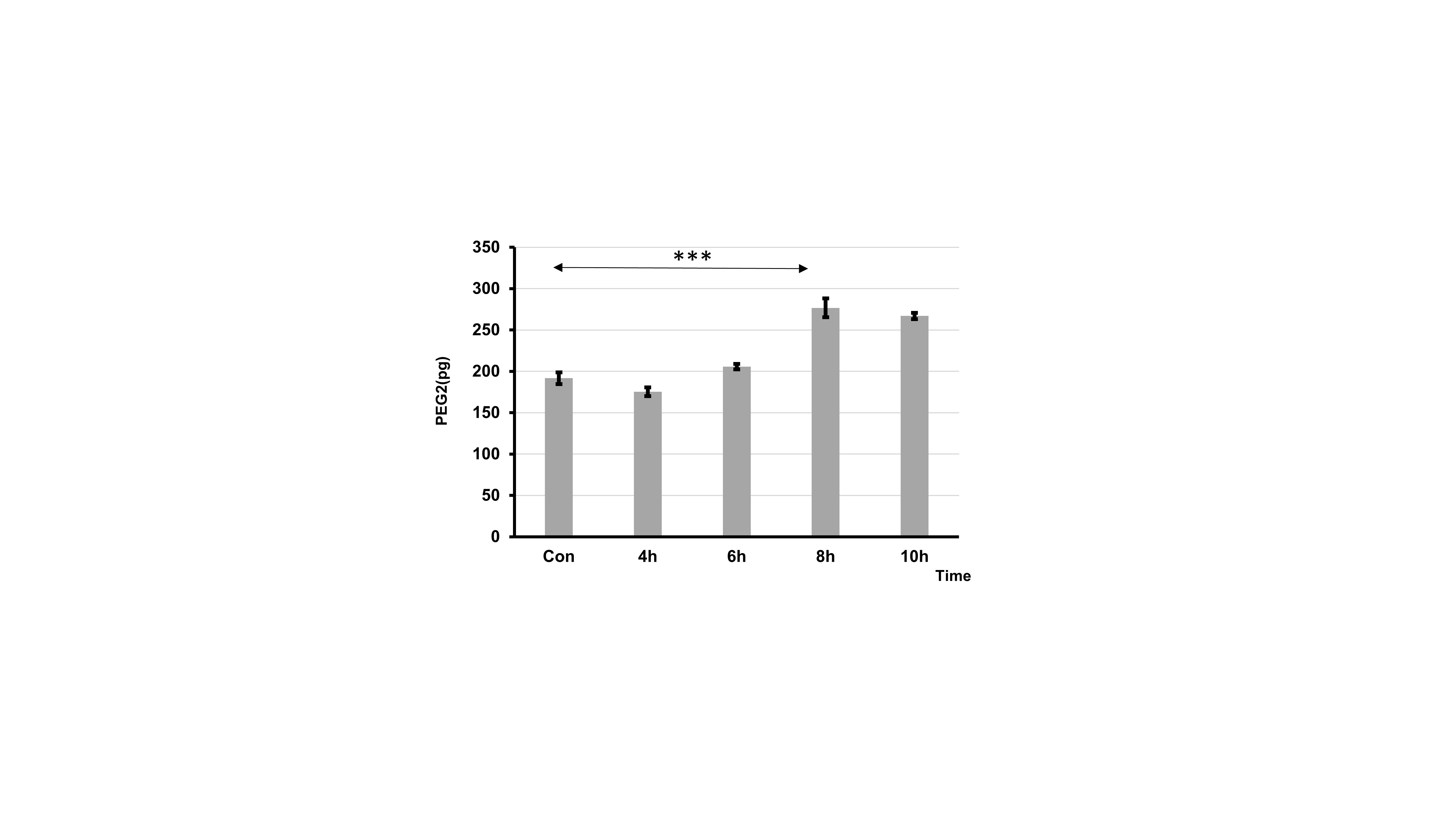


Fig.S4 Release of PEG2 from IC-SoC in response to stimulation (n = 5 per condition, p**<0.01; p***<0.001).


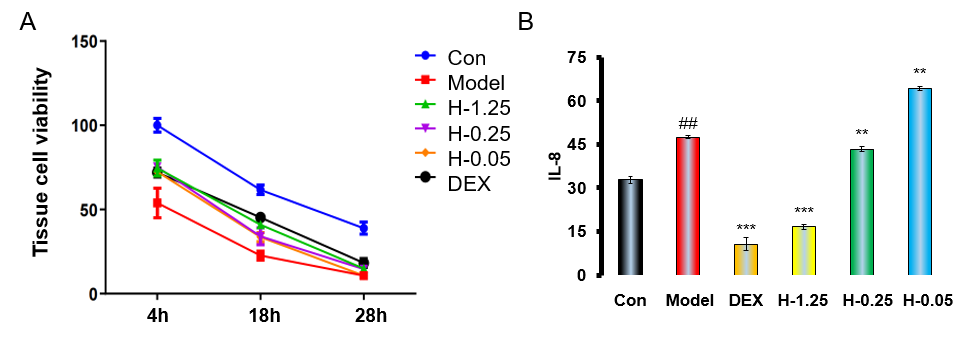


Fig.S5 Drug testing. (A) Cell viability in response to stimulation in control(Con) group, Model group (exposure to SLS+*P.acnes*) in different time during in response to stimulation) and DEX group (treated with dexamethasone for 24 hours after SLS+*P.acnes* stimulation for 4 hours) and H-1.25, H-0.25, H-0.05 (treated with 1.25, 0.25, 0.05 μM of Polyphyllin H for 24 hours after SLS+*P.acnes* stimulation for 4 hours). (B) Release of IL-8 in cultured HACAT cells in response to stimulation in Model group and DEX group and H-1.25, H-0.25, H-0.05 (n = 5 per condition, p**<0.01; p***<0.001).


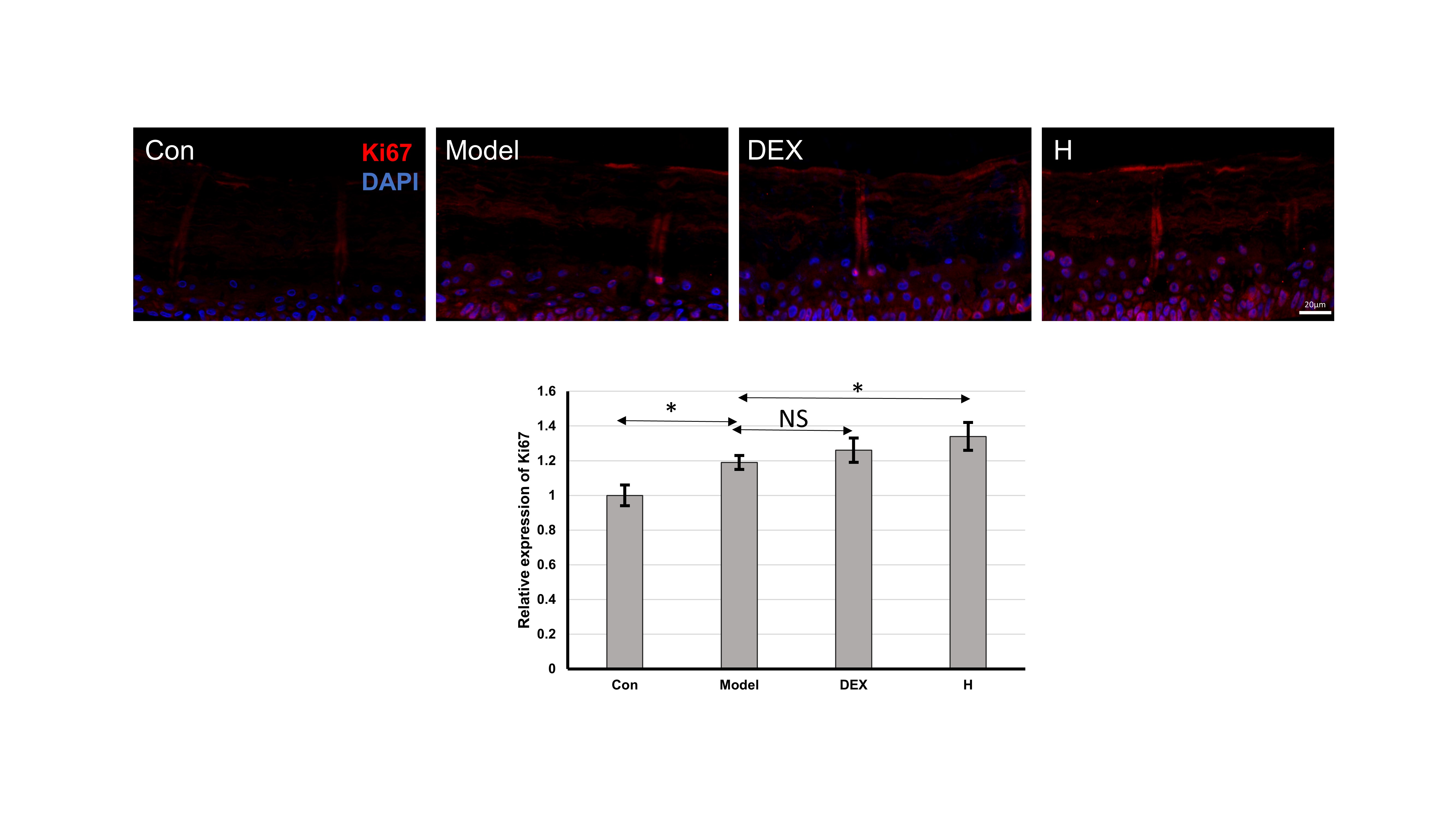


Fig.S6. Red Ki67 expression and blue DAPI for nuclei in different groups. Con (treated with PBS)，Model（treated with PBS for 24 hours after SLS+*P.acnes* stimulation for 4 hours) DEX (treated with dexamethasone for 24 hours after SLS+*P.acnes* stimulation for 4 hours) and H (treated with 1.25 μM Polyphyllin H for 24 hours after SLS+*P.acnes* stimulation for 4 hours). (n = 5 per condition, p*<0.05; p**<0.01; p***<0.001).
